# Effects of bed baths with weak wiping pressure using cotton and disposable towels on the skin barrier function of the lower limbs and forearms in older patients with heart disease: a quasi-experimental (crossover) study

**DOI:** 10.1186/s40101-026-00433-x

**Published:** 2026-04-30

**Authors:** Inaho Shishido, Rika Yano

**Affiliations:** Faculty of Health Sciences, Hokkaido University, Kita 12, Nishi 5, Kita-Ku, Sapporo, 060-0812 Japan

**Keywords:** Bed baths, Hygiene care, Lower limbs, Older patients, Skin care, Stratum corneum hydration, Transepidermal water loss

## Abstract

**Background:**

Bed baths are commonly used to maintain skin hygiene in older hospitalized patients whose skin may be affected by disease, treatment, and aging. The effects of bed baths on the skin barrier function, particularly in the lower limbs, in older patients with heart disease who may have fragile skin remain unclear. This quasi-experimental (crossover) study investigated the effects of bed baths with weak wiping pressure using cotton and disposable towels on the skin barrier function of the lower limbs and forearms in older hospitalized patients with heart disease.

**Methods:**

Thirty-three older hospitalized patients with heart disease were evaluated. Participants received two randomly ordered wipes (AB or BA): (A) bed baths of the lower limbs and forearms using disposable towels; and (B) bed baths of the lower limbs and forearms using cotton towels. Weak wiping pressure was used (10–20 mmHg). Skin barrier function was measured before, 15 min after, and the day after bed bath using transepidermal water loss (TEWL), stratum corneum hydration (SCH), and overall dry skin score (ODS). Mixed-effects models for repeated measures were used to compare the changes over time between the two types of bed baths.

**Results:**

SCH and TEWL were lower in the lower limbs than in the forearms, while ODS was higher. An interaction for TEWL was observed in the lower limbs (*F*_[2__,25__]_ = 4.0, *P* = 0.030); however, TEWL did not differ significantly across time points or towel types. No interaction or main effects of time or towel type on lower limb SCH and forearm TEWL were noted. Only the main effect of time on forearm SCH was observed, which was significantly lower 15 min after the cotton towel bed bath than before (*t* = 3.2, *P* = 0.004, MD [95% CI]: − 3.5, − 0.8). ODS at baseline and the following day demonstrated no difference.

**Conclusion:**

Changes in TEWL and SCH over time revealed that a single bed bath with weak wiping pressure did not cause sustained impairment of skin barrier function by the following day. However, transient SCH decreases, and limited intervention periods require further mechanistic investigation.

**Trial registration:**

UMIN R000061354 (date of registration: March 3, 2024).

**Supplementary Information:**

The online version contains supplementary material available at 10.1186/s40101-026-00433-x.

## Background

Skin hygiene is essential for maintaining skin integrity and overall health [1]. Patients who are bedridden or have limited self-care abilities due to physical or mental impairments often receive bed baths to maintain skin hygiene [2]. Typically, hot towels are used to wipe the skin during bed baths. Optimal skin care should maintain cleanliness and skin integrity while providing comfort to the patients [3]. Due to the aging of the global population, many hospitalized patients are older adults, who comprise the majority of those receiving bed baths. As older patients tend to have fragile skin due to age-related changes [4–6], it is important to consider the effects of bed baths on their skin barrier function.

Regarding the effects of bed baths on the skin barrier function, a literature review identified frictional stimulation and chemical products as the key influencing factors [7]. Frictional stimulation of the skin during bed bathing is influenced by both the wiping pressure and towel material. Konya et al. [8, 9] classified wiping pressure into weak (10–20 mmHg) and ordinary (20–30 mmHg) based on a survey conducted among 101 nurses. Weak wiping pressure did not impair the skin barrier function of the forearms in older patients [10, 11]. Furthermore, the safety of weak wiping pressure on the forearm has been verified even in older patients with dry skin conditions that require special skin care [11]. Matsumoto et al. [12] reported that disposable towels were more effective than cotton towels for maintaining stratum corneum hydration (SCH) on the inner forearm and thigh after bed baths in healthy older adults. Schoonhoven et al. [13] found that six weeks of bed baths using disposable towels slightly reduced the number of skin abnormalities on the buttocks, abdomen, and groin compared to cotton towels, although there was no significant difference in skin lesions. As such, evidence is accumulating regarding the impact of bed baths on the skin barrier function. Notably, many studies selected the flexor side of the forearm as the target site, as it has less body hair and surface irregularities, making the measurement of the skin barrier function easier and reliable. However, dry skin is commonly observed on the extremities, with a higher incidence on the lower limbs than on the forearms [14, 15]. In addition, the lower limbs are more prone to skin tears and edema [16]. Therefore, the skin barrier function in the lower limbs is more prone to deterioration than in other areas, and special care is required when providing bed baths.

To our knowledge, only one study [17] has evaluated the effect of bed baths on the skin barrier function of the lower limbs. In that study [17], bed baths of the lower limbs with disposable or cotton towels were performed for 12 weeks, and changes in SCH before and after the bed baths were compared. However, transepidermal water loss (TEWL) was not measured, which is an important parameter for measuring the skin barrier function [18]. The primary skin barrier is located in the stratum corneum [19]. TEWL is defined as the flux density of water diffusing from the dermis and epidermis through the stratum corneum to the skin surface [20] and is directly correlated with skin barrier dysfunction [21]. The SCH reflects the water content of the stratum corneum. Therefore, the effects of bed baths on the skin barrier function of the lower limbs have not been fully investigated yet.

Older patients with heart disease often receive bed baths to maintain skin cleanliness due to their unstable circulation and difficulty in showering or bathing. In a previous study [22], older patients with heart disease were identified as the target population who need appropriate skin care after careful consideration of their skin condition. In addition to age-related changes in the skin, these patients often take medications that increase the risk of skin dryness, such as diuretics [23] and statins [24], and frequently have comorbid conditions such as chronic kidney disease and diabetes [24, 25]. Furthermore, due to the existence of disease, they often experience edema caused by fluid retention, making them a population in whom changes in the skin barrier function of the lower limbs during bed bathing should be monitored carefully. However, evidence regarding the skin barrier function in patients with heart disease remains limited, and it is necessary to examine how bed baths for older patients with heart disease affect the skin barrier function of the limbs, including the lower limbs.

This study aimed to examine the effects of bed baths with weak wiping pressure using cotton and disposable towels on the skin barrier function of the lower limbs and forearms in older hospitalized patients with heart disease. This study sought to test the following hypotheses:

- Hypothesis 1: Bed baths with weak wiping pressure using disposable and cotton towels in older patients with heart disease negatively affect the skin barrier function of the lower limbs (TEWL increases and SCH decreases after bed baths compared to the values before the intervention).
- Hypothesis 2: Bed baths with weak wiping pressure using disposable and cotton towels in older patients with heart disease negatively affect the skin barrier function of the forearms (TEWL increases and SCH decreases after bed baths compared to the values before the intervention).

## Methods

### Study design and setting

This quasi-experimental crossover study was conducted between March and May 2024 (spring). To investigate the effect of towel materials on the skin barrier function, we used cotton and disposable towels, which are commonly used in clinical practice. Patients with heart disease received bed baths with disposable or cotton towels on either side of their forearms and lower limbs, and cotton or disposable towels on the opposite side. The order, as well as the left and right sides of the intervention, was randomly assigned. If a peripheral intravenous catheter or shunt for hemodialysis was present in the forearms or lower limbs, a bed bath was performed on the same side, with a minimum bed bathing interval of 48 h. The protocol applied to these patients was based on a previous study [11]. As with the other participants, we ensured an interval of more than 24 h between the skin barrier function measurements and any skin care procedures, including bed baths and moisturizer application, to minimize the impact on the measurements. The study was registered in the University Hospital Medical Information Network (registration no.: UMINR 000061354) and was performed according to the Transparent Reporting of Evaluations with Non-randomized Designs guidelines (TREND guidelines, Additional file 1) [26].

### Participants

This study included 33 hospitalized patients with heart disease at a hospital in northern Japan. The inclusion criteria were as follows: (1) patients aged 65 years or older and (2) those with heart disease. The exclusion criteria were as follows: (1) patients with difficulty in communication (e.g., dementia, delirium, or severe conditions) and (2) those who had been diagnosed with a skin disease or prescribed ointments or creams. Nursing managers in each unit identified eligible patients, and the researchers provided both verbal and written explanations of the study. Only those who gave written informed consent were included. Sample size calculations for the main analysis interaction were performed using the G Power software ver. 3.1.9 [27], assuming a median effect size of *η*^2^ = 0.07 [11], *α* = 0.05, and 1 − β = 0.80. The calculated minimum sample size was 28, and after accounting for a 20% dropout rate, 33 patients were included.

### Intervention and measurement areas

The target areas were both lower limbs, which are the most common sites of dry skin and edema, and both forearms [14, 15], which were the intervention sites in previous studies [10, 11].

### Intervention

#### Cotton towels

A washcloth (32 cm × 32 cm, 33 g) was wrung out to a total weight of 88 ± 2 g, including water [28], and heated in a towel warmer (TW-7F, Daishin Shoji Ltd., Chiba, Japan) for 30 min to a surface temperature of approximately 43 °C. The weight of each towel was measured using a digital scale (KW-001; Tanita Ltd., Tokyo, Japan).

#### Disposable towels

A disposable towel (20 cm × 32 cm, 22 g, Mitsubishi paper mills Ltd., Tokyo, Japan) was heated in a towel warmer (TW-7F, Daishin Shoji Ltd., Chiba, Japan) for 15 min to a surface temperature of approximately 43 °C. The towels were made of water, propylene glycol, phenoxyethanol, benzalkonium chloride, and propynyl iodide butyl carbamate.

#### Bed baths

The interventions were performed on days without major invasive catheterization, treatment, surgery, bathing, or showering. In addition, participants were instructed to refrain from skin care procedures, including bed bathing, bathing, showering, and the application of moisturizers, from 24 h before the intervention until the day after the intervention.

The lower limb (or forearm) on either side was wiped with either a disposable towel or cotton towel thrice from the ankle to the knee joint on the front of the lower limb (or the forearm flexor, from the wrist to the elbow joint) [11, 28]. The moisture on the skin surface was wiped off with a dry towel (dry wiping). The contralateral side was similarly wiped with either a cotton or disposable towel, followed by dry wiping. There was no interval between the two types of bed baths because the interventions were performed on different body sites. The same researcher wiped each patient’s skin.

Wiping pressure during bed baths was set at 10–20 mmHg, based on a previous study [9] that showed that this low pressure effectively removes dirt without impairing the skin barrier function in older adults. To control frictional stimulation during wiping, patients were required to remain in a supine position. For lower limb wiping, the patients’ knee joint was extended, and the lower limb was externally rotated. For forearm wiping, patients were asked to abduct the forearm so that the dorsal side was in contact with the bed surface. If the participants experienced respiratory distress on lying down, they were placed in the fueler or sitting position, and the posture was standardized among the participants. To optimize the wiping pressure, we gave bed baths to five healthy adult females (mean age: 27.0 [5.9] years, body mass index [BMI]: 21.3 [1.8]) in the laboratory. The intraclass correlation coefficient (ICC) for intra-rater reliability of the wiping pressure on the forearm was 0.66 (95% confidence interval [CI]: 0.17–0.90). As the ICC was above 0.61, the reliability was considered adequate [29].

### Data collection (Fig. 1)

**Fig. 1:**
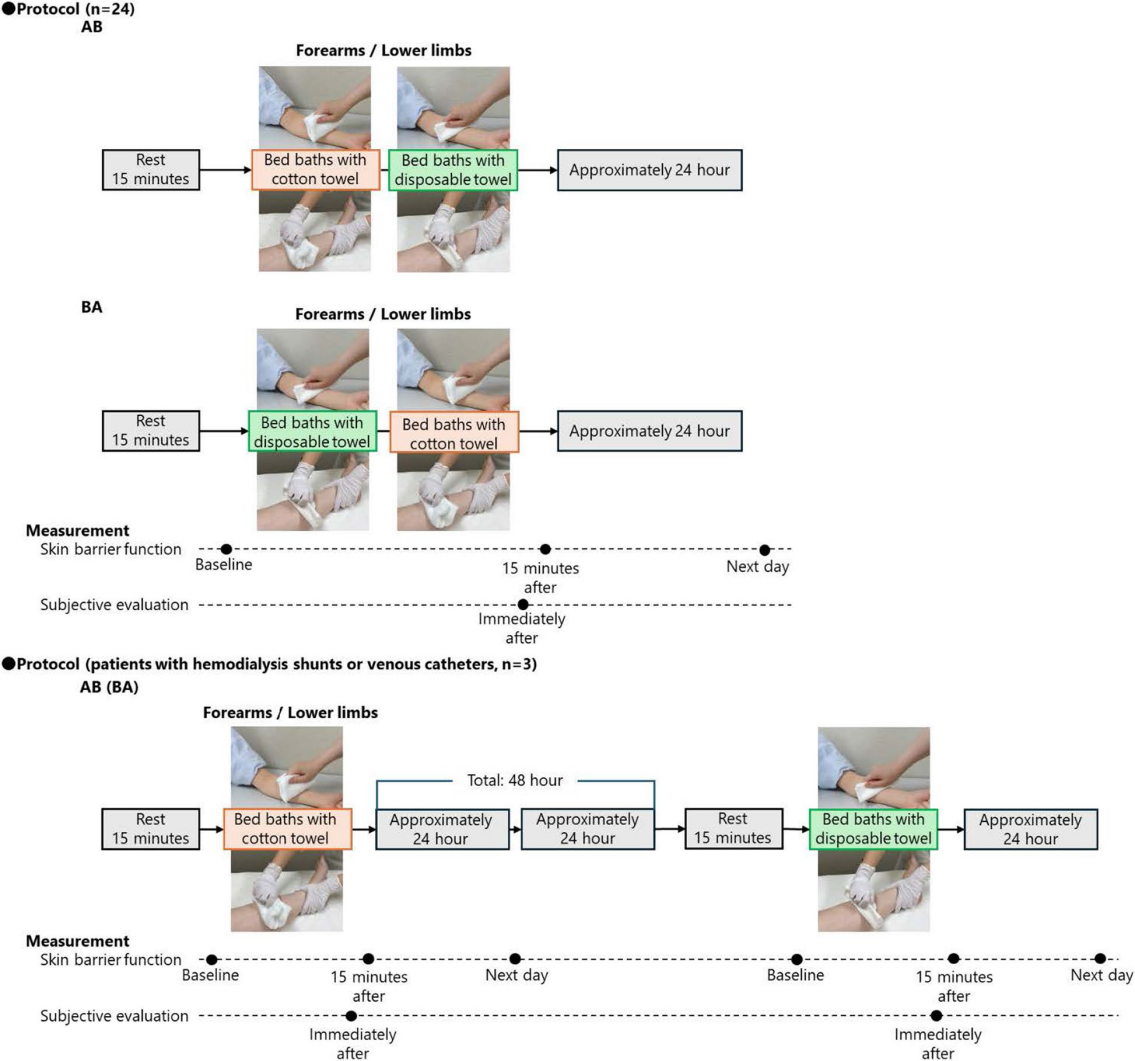
Study protocol. Notes: Black circles; measurements

### Participant characteristics

Patient information was collected from the nursing and electronic medical records, including demographic characteristics (age, sex, BMI [kg/m^2^]), hospitalization days, degree of independence (J, independent; A, house-bound; B, chair-bound; C, bed-bound), medication history (disease, medical history, and medical treatments [steroids, anticoagulants, diuretics, anticancer therapy, radiotherapy, and hemodialysis]), nutritional status, smoking history, and sun exposure. These items were selected based on a literature review that identified the factors associated with worsening skin barrier function in older patients [30]. Nutritional status was assessed using the controlling nutritional status (CONUT) score, which was automatically calculated from the serum albumin, total cholesterol, and total lymphocyte count [31]. In general, the nutritional status was assessed based on the BMI and dietary intake. However, participants in this study were prone to weight gain due to fluid retention as a result of heart and kidney failure, making it difficult to use only the BMI to assess the nutritional status. In addition, prehospitalization skin care habits, subjective skin symptoms, dryness, and edema status were assessed prior to the intervention. Edema of both the lower limbs and forearms was evaluated based on the presence and degree of indentation after pressure was applied to each site while the participant was lying on the bed (0, none; 1, slight indentation [2 mm] and normal contours; 2, deeper pit after pressing [4 mm] and fairly normal contour; 3, deep pit [6 mm] that persists for several seconds after pressing; and 4, deep pit [8 mm] that persists for a prolonged time, possibly minutes, after pressing) [32].

### Skin barrier function

**Fig. 2:**
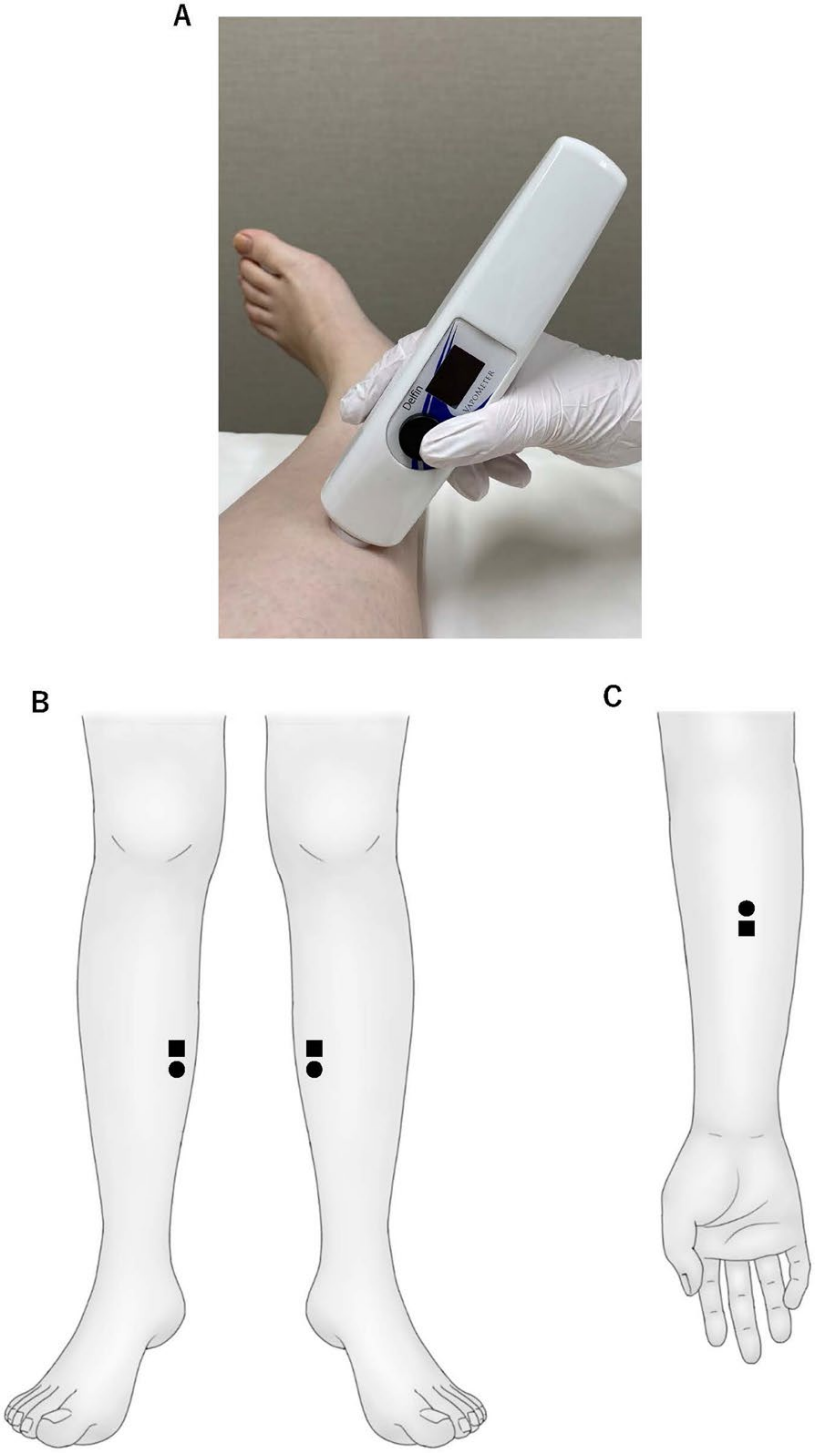
Measurement site for the skin barrier function. Notes: **A** measurements of skin barrier function; **B** the lower limbs; **C** the inner forearms. Squares, measurement site of transepidermal water loss; circles, stratum corneum hydration

The skin barrier function was evaluated on the anterior surface of the lower limbs and inner forearms before the bed bath (baseline), 15 min after the bed bath (15 min after), and the day after the bed bath (next day). The measurement time points were determined based on the protocols of previous studies [11, 28] with consideration given to avoiding the potential effects of meals or rehabilitation activities. The participants were asked to rest for 15 min before the skin barrier function was measured. To avoid measurement errors, it was ensured that uniform pressure was generated when the device touched the measurement site. The participants were asked to lie down to ensure stable probe contact with the skin. When measuring the skin barrier function of the lower limbs, the patients’ knee joint was extended, and the lower limb was externally rotated (Fig. 2a). The skin barrier function on the lower limbs was measured at the knee joint, medial to the tibia, and at the center of the ankle joint (Fig. 2b). The measurement sites in the flexed forearm were the elbow joint and the center of the wrist joint (Fig. 2c). Furthermore, the body hair and bone were avoided during measurements. If the participants experienced respiratory distress on lying down, they were placed in the fueler or sitting position, and the posture was standardized among the participants.

#### Trans-epidermal water loss (TEWL)

TEWL is the amount of water that passively evaporates through the skin to the external environment due to the water vapor pressure gradient on both sides of the skin barrier and is used to characterize the skin barrier function [33]. The higher the TEWL value, the greater the water loss and lower the skin barrier function [21]. TEWL was measured using a VapoMeter (SWL-5001; Delfin Technologies Ltd., Kuopio, Finland). The average of the 30 s values measured at each time point according to the guidelines [18] was used for further analysis.

#### Stratum corneum hydration (SCH)

SCH reflects the water content of the stratum corneum [18] and was measured using MoistureMeter (SC Compact, Delfin Technologies Ltd., Kuopio, Finland). Three measurements were performed at each point, and the average value was used for analysis. To minimize the influence on the TEWL measurement, SCH measurements were performed after the TEWL measurement [34].

In order to control the measurements, we measured TEWL and SCH in five healthy adult females (mean age 31.0 [3.6], BMI 19.7 [1.5]) in the laboratory. In the forearm, the ICCs for TEWL and SCH were 0.93 (95% CI: 0.70–0.98) and 0.96 (95% CI: 0.85–0.99), respectively. In the lower limbs, the ICCs for TEWL and SCH were 0.93 (95% CI: 0.77–0.98) and 0.98 (95% CI: 0.94–1.00), respectively. The ICCs were greater than 0.61, indicating adequate reliability [29].

#### Dry skin

Dry skin on the lower limbs and forearms was assessed using the Japanese version of the overall dry skin score (ODS) [35]. This scale is a typical skin assessment index that evaluates the presence and severity of skin dryness using a five-point scale (0, no skin dryness; 4, severe skin irritation, scale, inflammation, and cracking). The validity of this scoring system has been demonstrated [36].

### Subjective evaluation

Participants were asked to freely share their impressions of the bed bath at the end of each intervention. The specific questions were as follows: (1) How did you feel about receiving the bed bath? (2) Which type of bed bath did you prefer? To accurately record the participants’ response, an integrated circuit (IC) recorder was used to record the participants’ voices from the beginning to the end of each intervention.

### Participants’ clothing

Participants wore hospital gowns over undergarments. To ensure their comfort and prevent them from feeling cold, only the relevant body areas were exposed during the intervention and measurement of skin barrier function.

### Experimental procedure

Considering the burden placed on the participants, data collection was conducted in a hospital room. The room temperature and humidity were measured at all measurement points. The target temperature was set at 22–25 °C and the target humidity at 45–55% to standardize the measurement conditions. However, the actual room temperature (mean ± standard deviation) was 25.9 ± 1.2 °C before the intervention, 26.3 ± 1.2 °C after 15 min, and 25.0 ± 1.4 °C on the next day. The humidity (mean ± standard deviation) was 30.6 ± 6.7% before the intervention, 30.2 ± 6.7% after 15 min, and 31.4 ± 6.5% on the next day.

### Statistical analysis

For the participant characteristics, the mean and standard deviation or frequency were calculated and summarized. The skin barrier function was analyzed using mixed-effects models for repeated measures (MMRM), where time and towels were defined as fixed factors, whereas the participants were defined as random factors. The factors in this MMRM were “time” (baseline, 15 min after, and next day) and “towels” (disposable and cotton). For the interaction, “time and towels” was set up. If at least one of the factors or interactions was significant, a post hoc test was performed on the means to compare the data between the two intervention types. This study employed a crossover design, and variables such as the nutritional status, chronic kidney disease (CKD), and medications were not included as covariates. In addition, changes in the skin barrier function over time were compared using MMRM only in patients with dry skin and CKD, which are risk factors for skin barrier disorders [30]. The significance level was set at 5%. In this study, the Bonferroni method was used for the post-hoc test to confirm the differences between the time points within each condition and between the conditions at each time point (total of nine conditions); the *P*-value was determined to be significant at 0.05/9 = 0.0056. Analyses were conducted using JMP® Student Edition 18.2.1 (SAS Institute Inc., Cary, NC, USA). For the qualitative data analysis, the voice recordings captured on an IC recorder were transcribed verbatim, and the content was organized for thematic interpretation. Inter-rater reliability was evaluated using the intraclass correlation coefficient [ICC (1,1)], based on a one-way random-effects model with single measurements, calculated using the *irr* package in R version 4.5.2.

## Results

### Participant characteristics

**Fig. 3:**
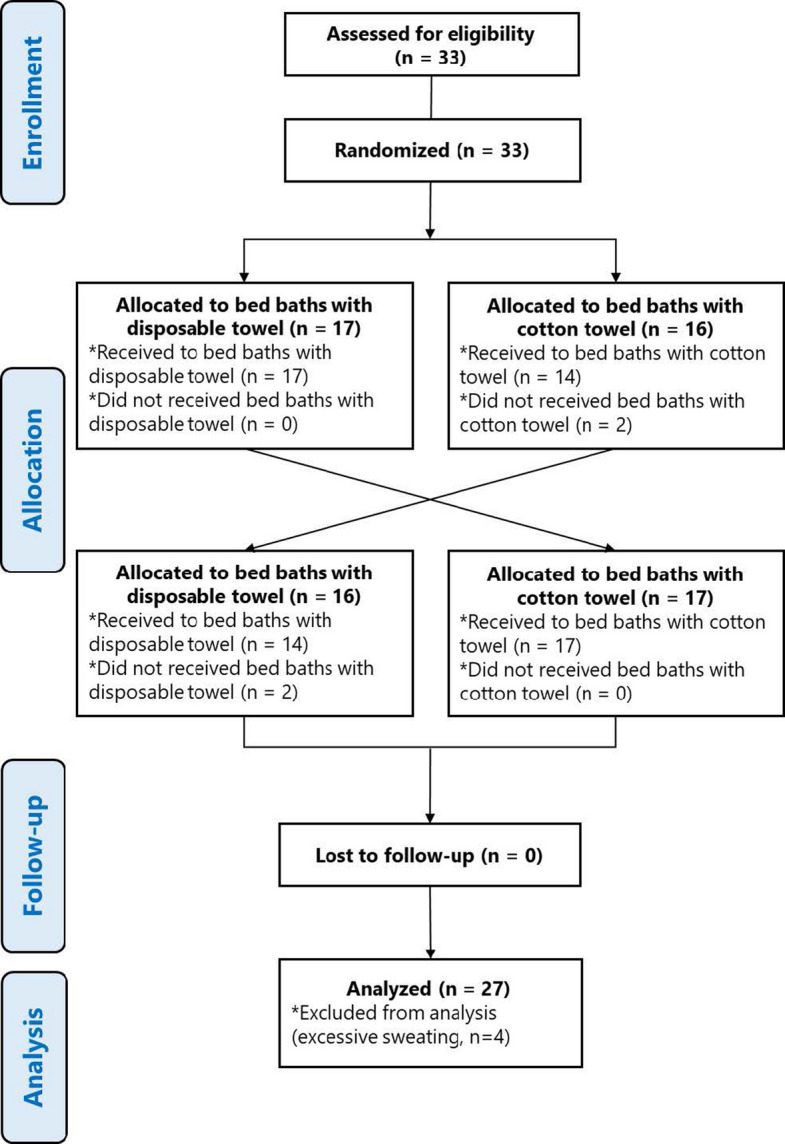
Diagram of the participant flow

The study initially enrolled 33 participants; however, two individuals were excluded because their physical condition or changes in treatment schedules prevented them from undergoing the bed bath, and four were excluded due to excessive sweating. Consequently, 27 participants were included in the analysis (Fig. 3). Table 1 presents the characteristics and baseline skin barrier function of the participants who were not included as well as the same parameter of those who were included in the final analysis. The TEWL values of the lower limbs and forearms were higher in participants who were not included in the final analysis than in those who were included. Moreover, participants who were excluded from the final analysis displayed a significantly higher number of showers per week compared to those who were included (Table 1). Of these, 16 (59.3%) were female and the mean age (standard deviation) was 83.1 (8.0) years. Twenty-five patients had dry skin (ODS ≥ 1) on the lower limbs and 23 had dry skin on the forearms. The mean ODS of the lower limbs (1.7 [0.7]) was significantly higher than that of the forearms (1.2 [0.7]) (*P* = 0.001, *t* = 3.5, mean difference [MD, 95% CI]: 0.2, 0.8). The mean TEWL (*P* = 0.008, *t* = − 2.7, MD [95% CI]: − 1.8, − 0.3) and SCH (*P* < 0.001, *t* = − 7.0, MD [95% CI]: − 7.1, − 4.0) of the lower limbs were significantly lower than those of the forearms (Table 1).

**Table 1:** Baseline characteristics of completers and dropouts

Variables		Completed group (*n* = 27) Values, range		Dropout group (*n* = 6) Values, range		MD (95% CI)	OR (95% CI)	*P* ^a^
**Sex [female]**	*n* (%)	16 (59.3)		4 (66.7)			0.7 (0.1, 4.7)	1.000
**Age**	years	83.1 (8.0)	67–99	80.5 (7.2)	67–88	− 2.6 (− 9.9, 4.7)		.471
**Body mass index**	kg/m^2^	20.8 (4.1)	14.2–28.7	23.4 (4.2)	17.4–29.4	2.6 (− 1.2, 6.4)		.177
**Duration of hospitalization**	days	12.7 (12.2)	2–51	3.5 (1.9)	2–7	− 9.2 (− 19.5, 1.2)		.080
**Disease**								
Myocardial ischemia	*n* (%)	11 (40.7)		4 (80.0) ^*1^			0.2 (0.0, 1.8)	.161
Heart valve diseases	*n* (%)	4 (14.8)		2 (40.0) ^*1^			0.3 (0.0, 2.1)	.228
Chagas cardiomyopathy	*n* (%)	5 (18.5)		0 (0.0) ^*1^			-	.564
Arrhythmias, cardiac	*n* (%)	18 (66.7)		4 (80.8) ^*1^			0.5 (0.0, 5.2)	1.000
Heart failure	*n* (%)	19 (70.4)		3 (60.0) ^*1^			1.5 (0.2, 11.4)	.637
Kidney failure	*n* (%)	9 (33.3)		2 (40.0) ^*1^			0.8 (0.1, 5.3)	1.000
Hypertension	*n* (%)	14 (51.9)		4 (80.0) ^*1^			0.3 (0.0, 2.7)	.355
Diabetes mellitus	*n* (%)	10 (37.0)		0 (0.0) ^*1^			-	.155
**Treatment**								
Renal dialysis	*n* (%)	1 (3.7)		0 (0.0)			-	1.000
Use of diuretics	*n* (%)	21 (77.8)		4 (66.7)			1.8 (0.3, 12.0)	.616
Use of antithrombic drug	*n* (%)	20 (74.1)		5 (83.3)			0.6 (0.1, 5.8)	1.000
Use of statin	*n* (%)	9 (33.3)		5 (83.3)			0.1 (0.0, 1.0)	.062
Use of steroid	*n* (%)	1 (3.7)		0 (0.0)			-	1.000
**Undernutrition degree**								
Normal (0–1)	*n* (%)	4 (14.8)		1 (16.7)			-	.903 ^b^
Light (2–4)	*n* (%)	5 (18.5)		2 (33.3)				
Moderate (5–8)	*n* (%)	15 (55.6)		3 (50.0)				
Severe (9–12)	*n* (%)	3 (11.1)		0 (0.0)				
**Smoking**	*n* (%)	11 (40.7)		2 (33.3)			0.7 (0.1, 4.7)	1.000
**Sunlight exposure**	*n* (%)	4 (14.8)		2 (33.3)			2.2 (0.3, 15.5)	.584
**Degree of independence**								
J, Independence	*n* (%)	12 (44.4)		4 (66.7)			-	.767 ^b^
A, Requires assistance to leave home	*n* (%)	7 (25.9)		2 (33.3)				
B, Nearly bedridden	*n* (%)	3 (11.1)		0 (0.0)				
C, Bedridden	*n* (%)	5 (18.5)		0 (0.0)				
**Overall dry skin score (ODS)**								
** Forearm** ODS = 0 ODS = 1 ODS = 2 ODS = 3	*n* (%)	4 (14.8) 14 (51.9) 9 (33.3) 0 (0.0)		1 (20.0) ^*1^ 3 (60.0) ^*1^ 1 (20.0) ^*1^ 0 (0.0) ^*1^			-	1.000 ^b^
** Lower limb** ODS = 0 ODS = 1 ODS = 2 ODS = 3	*n* (%)	2 (7.4) 7 (25.9) 16 (59.3) 2 (7.4)		1 (20.0) ^*1^ 1 (20.0) ^*1^ 3 (60.0) ^*1^ 0 (0.0) ^*1^			-	.792 ^b^
**Transepidermal water loss (TEWL) at baseline**								
Forearm	g/m^2^h	8.1 (2.5)	4.2–13.6 ^*2^	11.7 (5.2)	6.8–22.2 ^*3^	3.5 (1.4, 5.6)		**.001**
Lower limb	g/m^2^h	7.1 (2.0)	2.2–13.4 ^*2^	10.5 (6.5)	5.0–20.5 ^*3^	3.4 (1.2, 5.5)		**.003**
**Stratum corneum hydration (SCH) at baseline**								
Forearm	A.U.	17.9 (5.1)	10.1–31.0 ^*2^	16.9 (1.6)	11.0–26.7 ^*3^	− 1.0 (− 4.4, 2.5)		.578
Lower limb	A.U.	12.3 (4.9)	5.3–27.3 ^*2^	10.9 (1.5)	6.5–20.4 ^*3^	− 1.4 (− 4.7, 1.9)		.407
**Subjective skin symptoms**								
Feeling of itchy skin	*n* (%)	8 (29.6)		0 (0.0)			-	.296
Feeling of dry skin all the time	*n* (%)	12 (44.4)		1 (16.7)			-	.195 ^b^
Feeling of dry skin in winter	*n* (%)	7 (25.9)		3 (50.0)				
Feeling of dry skin after taking a bath	*n* (%)	1 (3.7)		0 (0.0)				
**Edema (Scale ≥ 1)**								
Forearm	*n* (%)	12 (44.4)		2 (40.0) ^*1^			1.2 (0.2, 8.4)	1.000
Lower limb	*n* (%)	13 (48.1)		3 (60.0) ^*1^			0.6 (0.1, 4.3)	1.000
**Skin care habits**								
Taking a bath	times/week	2.3 (1.9)	0–7	1.8 (1.9)	0–5	− 0.5 (− 2.2, 1.2)		.567
Taking a shower	times/week	0.4 (0.9)	0–3	1.7 (1.6)	0–4	1.3 (0.3, 2.3)		**.012**
Others	times/week	0.1 (0.7)	0–3.5	0.7 (1.6)	0–4	0.5 (− 0.3, 1.4)		.196
Scrubbing when washing	*n* (%)	10 (37.0)		2 (33.3)			1.2 (0.2, 7.6)	1.000
Using a lot of soap	*n* (%)	3 (11.1)		3 (50.0)			0.1 (0.0, 0.9)	.058
Lathering soap	*n* (%)	12 (44.4)		3 (50.0)			0.8 (0.1, 4.7)	1.000
Soaking in hot water	*n* (%)	6 (22.2)		0 (0.0)			-	.563
Wiping hard with a towel	*n* (%)	5 (18.5)		0 (0.0)			-	.556
Applying moisturizer	*n* (%)	2 (7.4)		1 (16.7)			0.4 (0.0, 5.3)	.464

*CI*, confidence interval; *MD*, mean difference; *OR*, odds ratio

*1, *n* = 5; *2, *n* = 54; *3, *n* = 10

^a^*t*-test or Fisher’s exact test; ^b^Freeman-Halton extension of Fisher’s exact test

Statistically significant values were marked in bold

### Change in the skin barrier function

**Fig. 4:**
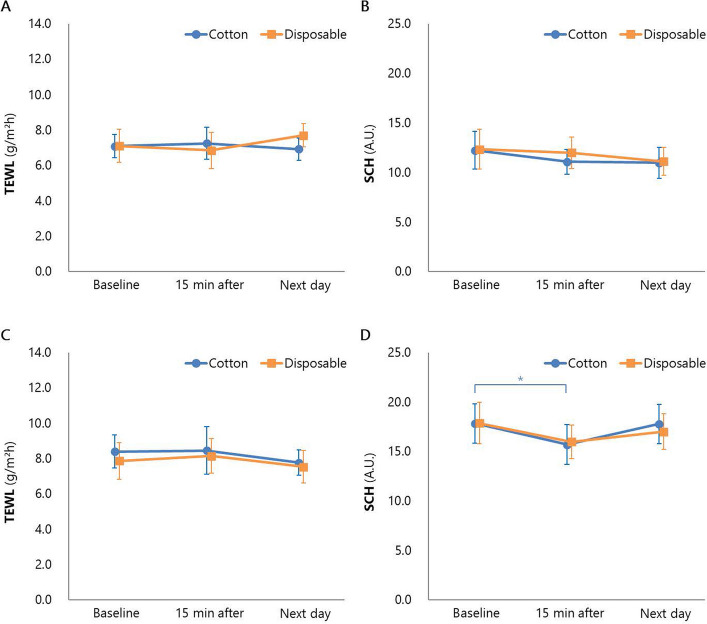
Skin barrier function changes over time in forearms and lower limbs by towel material (*n* = 27). Notes: **A** TEWL in the lower limbs; **B** SCH in the lower limbs; **C** TEWL in the inner forearms; **D** SCH in the inner forearms. Circles and squares, the least-square means as estimated by the mixed-effects model; error bar, 95% confidence interval. *P < 0.0056. SCH, stratum corneum hydration; TEWL, transepidermal water loss

Although the main effects of time (*F*_[2, 25]_ = 0.5, partial *η*^2^ = 0.04, *P* = 0.617) and towel type (*F*_[1, 26]_ = 0.3, partial *η*^2^ = 0.02, *P* = 0.592) on TEWL of the lower limbs were not significant, the interaction between time and towel type was significant (*F*_[2, 25]_ = 4.0, partial *η*^2^ = 0.24, *P* = 0.030). However, no differences were observed in the TEWL values of the lower limbs across the time points or towel types (Fig. 4A). The main effects of time (*F*_[2, 25]_ = 0.8, partial *η*^2^ = 0.06, *P* = 0.443) and towel type (*F*_[2, 25]_ = 2.3, partial *η*^2^ = 0.16, *P* = 0.120) on SCH of the lower limbs and the interaction (*F*_[1, 26]_ = 0.6, partial *η*^2^ = 0.05, *P* = 0.451) for SCH of the lower limbs were not significant (Fig. 4B). Although not statistically significant, the MD for SCH in lower limbs from baseline to the following day was − 1.3 A.U. (95% CI: − 2.8, 0.3) under the cotton towel condition and − 1.2 A.U. (95% CI: − 2.5, 0.0) under the disposable towel condition (Table 2).

**Table 2:** Details of statistical information in Fig. 4

	**T1**		**T2**		**T3**		***T*-test**								
	**Baseline**		**15 min after**		**Next day**		**T2-T1**			**T3-T1**			**T3-T2**		
	**M**	**95% CI**	**M**	**95% CI**	**M**	**95% CI**	**MD**	**95% CI**	***p***	**MD**	**95% CI**	***p***	**MD**	**95% CI**	***p***
**Lower limb (*n* = 27)**															
** TEWL**															
Cotton towel	7.1	6.4, 7.8	7.2	6.3, 8.2	6.9	6.3, 7.5	0.2	*−*0.7, 1.2	.709	*−*0.2	*−*0.7, 0.4	.510	*−*0.3	*−*1.1, 0.4	.392
Disposable towel	7.1	6.2, 8.0	6.9	5.8, 7.9	7.7	7.1, 8.4	*−*0.3	*−*1.2, 0.7	.586	0.6	*−*0.2, 1.4	.139	0.9	*−*0.0, 1.7	.050
** SCH**															
Cotton towel	12.2	10.3, 14.1	11.1	9.8, 12.4	11.0	9.4, 12.5	*−*1.2	*−*2.4, 0.1	.073	*−*1.3	*−*2.8, 0.3	.102	*−*0.1	*−*1.4, 1.1	.858
Disposable towel	12.4	10.3, 14.4	12.0	10.4, 13.6	11.1	9.7, 12.5	*−*0.4	*−*1.5, 0.8	.523	*−*1.2	*−*2.5, 0.0	.055	*−*0.9	*−*2.1, 0.4	.166
**Inner forearm (*n* = 27)**															
** TEWL**															
Cotton towel	8.4	7.5, 9.3	8.4	7.1, 9.8	7.8	7.1, 8.5	0.1	*−*1.3, 1.4	.936	*−*0.6	*−*1.6, 0.3	.193	*−*0.7	*−*2.0, 0.7	.307
Disposable towel	7.9	6.8, 8.9	8.1	7.2, 9.1	7.5	6.6, 8.4	0.3	*−*1.0 1.6	.655	*−*0.3	*−*1.1, 0.4	.361	*−*0.6	*−*1.7, 0.5	.247
**SCH**															
Cotton towel	17.8	15.9, 19.8	15.7	13.7, 17.7	17.8	15.8, 19.8	*−*2.2	*−*3.5, *−*0.8	**.004**	*−*0.1	*−*1.1, 1.0	.908	2.1	0.6, 3.6	.007
Disposable towel	17.9	15.8, 19.9	16.0	14.3, 17.7	17.0	15.2, 18.8	*−*1.9	*−*3.2, *−*0.6	.008	*−*0.9	−2.1, 0.3	.148	1.0	0.1, 1.9	.026

*CI*, confidence interval; *M*, the least square means as estimated by the mixed model; *MD*, mean difference; *SCH*, stratum corneum hydration; *TEWL*, transepidermal water loss

Statistically significant values were marked in bold

There was no significant interaction (*F*_[2, 25]_ = 0.2, partial *η*^2^ = 0.02, *P* = 0.806) and main effects of time (*F*_[2, 25]_ = 1.4, partial *η*^2^ = 0.10, *P* = 0.271) and towel type (*F*_[1, 26]_ = 1.4, partial *η*^2^ = 0.10, *P* = 0.255) on TEWL of the forearms (Fig. 4C). The interaction (*F*_[2, 25]_ = 1.1, partial *η*^2^ = 0.08, *P* = 0.339) and main effect of towel type (*F*_[1, 26]_ = 0.0, partial *η*^2^ < 0.01, *P* = 0.837) on SCH of the forearms were not significant, but the main effect of time was significant (*F*_[2, 25]_ = 8.8, partial *η*^2^ = 0.40, *P* = 0.001). For bed baths with cotton towels, SCH at 15 min after the intervention was significantly lower than the SCH at baseline (*t* = 3.2, *P* = 0.004, MD [95% CI]: − 3.5, − 0.8) (Fig. 4D).

### Changes over time in the skin barrier function of the lower limbs and forearms by towel material in patients with dry skin

**Fig. 5:**
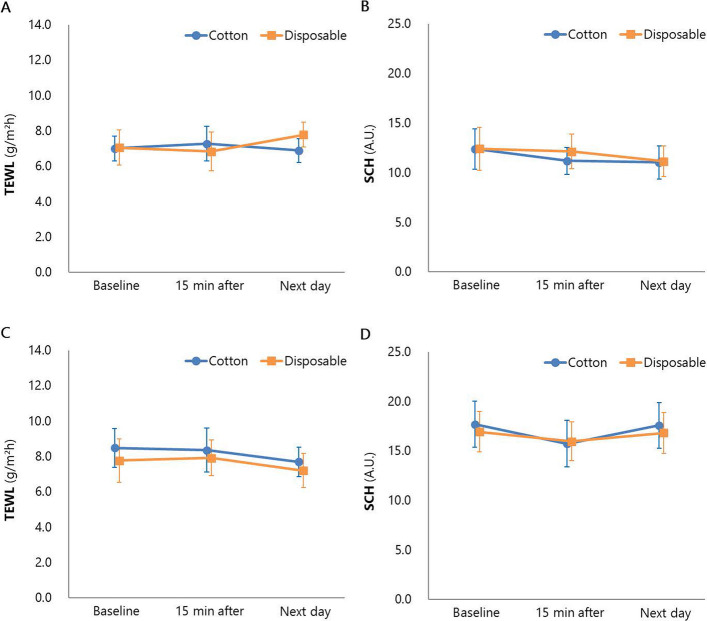
Skin barrier function changes in patients with dry skin (lower limbs: *n* = 25; inner forearms: *n* = 23). Notes: **A** TEWL in the lower limbs; **B** SCH in the lower limbs; **C** TEWL in the inner forearms; **D** SCH in the inner forearms. Circles and squares, the least-square means as estimated by the mixed model; error bar, 95% confidence interval. SCH, stratum corneum hydration; TEWL, transepidermal water loss

In patients with dry skin (ODS ≥ 1) on the lower limbs (*n* = 25), the main effect of time (*F*_[2, 23]_ = 0.8, partial *η*^2^ = 0.06, *P* = 0.477) and towel type (*F*_[1, 24]_ = 0.4, partial *η*^2^ = 0.02, *P* = 0.528) on TEWL of the lower limbs were not significant, although the interaction between time and towel type was significant (*F*_[2, 23]_ = 4.6, partial *η*^2^ = 0.29, *P* = 0.021). However, there were no differences in the TEWL values of the lower limbs across the time points or towel types (Fig. 5A). The main effects of time (*F*_[2, 23]_ = 0.9, partial *η*^2^ = 0.07, *P* = 0.439) and towel type (*F*_[2, 23]_ = 2.1, partial *η*^2^ = 0.16, *P* = 0.141) on SCH of the lower limbs and the interaction (*F*_[1, 24]_ = 0.5, partial *η*^2^ = 0.02, *P* = 0.508) for SCH were not significant (Fig. 5B).

In contrast, in patients with skin dryness on the forearms (n = 23), there were no significant interaction (*F*_[2, 21]_ = 0.1, partial η^2^ = 0.01, *P* = 0.910) and main effects of time (*F*_[2, 21]_ = 1.7, partial η^2^ = 0.14, *P* = 0.215) and towel type (*F*_[1, 22]_ = 3.1, partial η^2^ = 0.12, *P* = 0.093) on TEWL of the forearms (Fig. 5C). The interaction (*F* _[2, 21]_ = 0.7, partial η^2^ = 0.07, *P* = 0.495) and main effect of towel type (*F*_[1, 22]_ = 0.3, partial η^2^ = 0.01, *P* = 0.618) on SCH of the forearms were not significant, but the main effect of time was significant (*F*_[2, 21]_ = 5.3, partial η^2^ = 0.34, *P* = 0.013). However, there were no significant differences in the SCH measurements of the forearms across time points (Fig. 5D).

### Changes over time in the skin barrier function of the lower limbs and forearms by towel material in patients with CKD (n = 9)

**Fig. 6:**
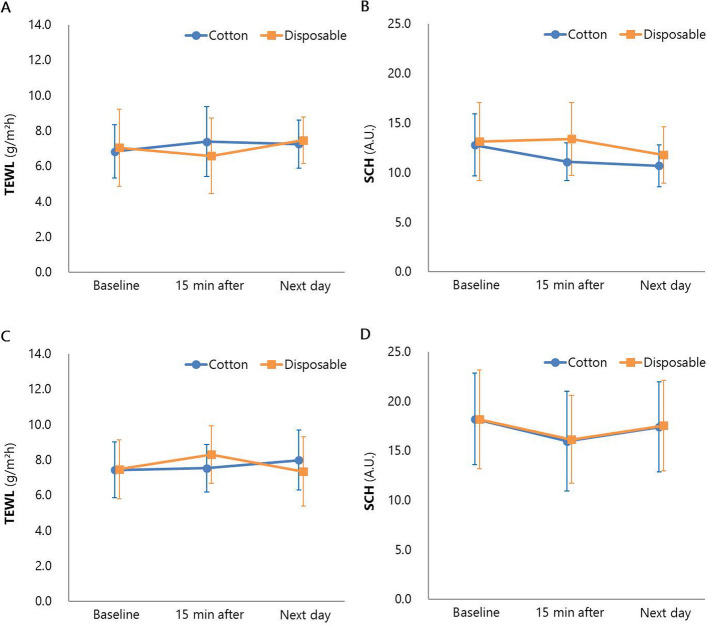
Changes over time in skin barrier function by towel material in patients with CKD (*n* = 9). Notes: **A** TEWL in the lower limbs; **B** SCH in the lower limbs; **C** TEWL in the inner forearms; **D** SCH in the inner forearms. Circles and squares, the least-square means as estimated by the mixed model; error bar, 95% confidence interval. SCH, stratum corneum hydration; TEWL, transepidermal water loss; CKD, chronic kidney disease

No interaction (*F*_[2, 7]_ = 0.9, partial *η*^2^ = 0.20, *P* = 0.444) or main effects of time or towel material (time, *F*_[2, 7]_ = 0.3, partial *η*^2^ = 0.08, *P* = 0.750; towel, *F*_[1, 8]_ = 0.1, partial *η*^2^ = 0.01, *P* = 0.750) were observed for TEWL of the lower limbs of patients with CKD (Fig. 6A). Furthermore, no interaction (*F*_[2, 7]_ = 1.0, partial *η*^2^ = 0.22, *P* = 0.429) or main effects (time, *F*_[2, 7]_ = 2.7, partial *η*^2^ = 0.44, *P* = 0.131; towel, *F*_[1, 8]_ = 1.2, partial *η*^2^ = 0.13, *P* = 0.313) on SCH of the lower limbs were observed (Fig. 6B). Similarly, no interaction or main effects on TEWL (interaction, *F*_[2, 7]_ = 1.1, partial *η*^2^ = 0.23, *P* = 0.399; main effect of time,* F*_[2, 7]_ = 0.3, partial *η*^2^ = 0.09, *P* = 0.731; main effect of towel, *F*_[1, 8]_ < 0.0, partial *η*^2^ < 0.01, *P* = 0.885) and SCH (interaction,* F*_[2, 7]_ < 0.0, partial *η*^2^ = 0.01, *P* = 0.966; main effect of time,* F*_[2, 7]_ = 2.8, partial *η*^2^ = 0.44, *P* = 0.130; main effect of towel, *F*_[1, 8]_ < 0.0, partial *η*^2^ < 0.01, *P* = 0.945) were observed in the forearms (Fig. 6C, 6D).

### Subjective evaluation

There were no complaints of pain or desire to not use either of the towels, and 11 patients (40.7%) described the wiping as pleasant during and after the bed bath. For bed bath of the lower limbs, 14 patients (51.9%) responded that either towel option was acceptable, nine (33.3%) preferred disposable towels, and four (14.8%) preferred cotton towels. For bed bath of the forearms, 15 (55.6%) responded that either option was acceptable, seven (25.9%) preferred disposable towels, and five (18.5%) preferred cotton towels. The impressions of bed bath with disposable towel were “feels soft (n = 5),” “feels smooth (*n *= 3),” and “feels fresh (*n* = 1).” For cotton towels, respondents commented that “cotton towels are rougher (*n* = 2),” “feel more familiar (*n* = 1),” and “thicker and more comfortable on the skin (*n* = 1).”

## Discussion

### Skin condition of older patients with heart disease

The prevalence of dry skin among the patients in this study was approximately 90%, higher than that in previous studies (Konya et al., 2021 [10], 64.3% [inner forearms]; 79.5–88.6% [lower limbs]; Yao et al., 2023 [5], 53% [95% CI: 36–69%]). The first reason for the tendency toward dry skin among the study participants may be that many of them had CKD [30] or were taking diuretics [23], which are associated with skin barrier dysfunction and dryness. Heart disease and kidney failure affect the systemic blood supply and lead to atrophy of the sweat and sebaceous glands. Therefore, despite the small number of bedridden patients included in this study, many participants had dry skin. Second, appropriate self‑care was not being practiced routinely. This is indicated by the fact that only 44% of the participants reported having dry skin, as well as by the presence of individuals who reported scrubbing their skin vigorously when washing and the very small number of participants who applied moisturizers. Previous studies [37] have reported that daily skin care habits are associated with dry skin. Thus, poor skin care habits may have contributed to the high prevalence of dry skin symptoms. Thirdly, seasonal factors may have played a role. To avoid the influence of perspiration on skin barrier function measurements, this study was conducted between March and May (spring), when humidity was low, around 30%. Previous research [38] has shown that the skin tends to be drier in winter than in summer. Therefore, it is likely that seasonal conditions also contributed to the high prevalence of dry skin observed in this study.

In addition, TEWL and SCH at baseline were lower in the lower limbs than in the forearms, while the ODS was higher in the lower limbs than in the forearms. Although impaired skin barrier function is generally characterized by increased TEWL and decreased SCH, TEWL may also be low when the skin is dry because there is less moisture available to evaporate. Based on these results, it was confirmed that the lower limbs were drier than the forearms. This trend observed in the older population is consistent with that reported in previous studies [15, 39, 40]. These findings confirm that the skin on the lower limbs in the study population requires particular attention with regard to the skin barrier function.

### Effects of bed baths with weak wiping pressure on the skin barrier function of the lower limbs and forearms in older hospitalized patients with heart disease

TEWL and SCH did not change significantly after bed baths of the lower limbs, along with the ODS. Moreover, these results were similar in participants with dry skin and CKD, which are risk factors for skin barrier dysfunction in older patients [30]. There were no significant differences in TEWL and SCH between bed baths using disposable and cotton towels 15 min after the bed bath and the next day. These results reject Hypothesis 1, that weak-pressure bed baths of the lower limbs using cotton towels or disposable towels in older patients with cardiac disease would reduce the skin barrier function. There were no negative opinions in the subjective evaluations, and the majority of respondents indicated that both towel materials were acceptable for both the lower limbs and forearms. These results confirm the safety of bed bathing with weak wiping pressure on the lower limbs. Therefore, selecting the towel materials according to the patient’s preference is considered appropriate for ensuring patient-centered care.

However, although there was no statistically significant difference, MD in SCH of the lower limbs between baseline and the day after bed bathing was 1.2–1.3, which is a greater change than the smallest detectable change (0.31 A.U.) reported in a previous study that assessed skin barrier function in older adults [11]. Similarly, in a study by Gillis et al. [17], unlike the temporal changes observed in forearm SCH after bed bathing, lower limbs SCH showed a slight downward trend following the bed bath, consistent with the findings of the present study. The forearm and lower limb skin contains less sebum compared with areas such as the face [41], and the lower limb is drier than the forearm. It has been shown that when skin dryness occurs, the skin’s inherent barrier function declines [42]. Since this study involved only a single intervention, we cannot postulate how the skin condition of the lower limbs might change with daily bed baths. Considering that bed bathing is routinely provided as a part of daily care, it is worth exploring how the skin condition of the lower limbs changes with continuous interventions in future studies.

Although no differences were observed in TEWL during bed bathing of the forearm with a cotton towel, SCH measured 15 min after the bed bath was significantly lower than that before the bed bath (*t* = 3.2, *P* = 0.004, MD [95% CI]: 0.8–3.5). Based on this finding, Hypothesis 2 was rejected. In a previous study in which bed bathing with weak wiping pressure was performed on the forearms of older adults [11], SCH on the forearm also decreased once after bed bathing and returned to baseline the following day. A possible explanation is that the warmth of the towel promoted evaporation of moisture from the stratum corneum. In particular, because the surface of a cotton towel has loops called piles and is therefore rougher than a disposable towel, it may have caused greater frictional stimulation of the skin. Moreover, the disposable towel contained moisturizing ingredients. These factors may have contributed to the decrease in SCH 15 min after bed bathing only with the cotton towel.

### Limitations

This study has several limitations. First, the room temperature at the measurement site was high and humidity was low. Because the interventions and evaluations of skin barrier function were conducted in hospital rooms, rather than in controlled laboratory settings, the room temperature and humidity may have influenced the measured values. Future studies should consider supplementing instrumental measurements with visual skin assessments, such as the ODS. Second, the sample size was small and limited to patients with heart diseases. Given that the skin integrity may be variably affected by different diseases and treatments, future studies should include more diverse patient populations, such as those with cancer. Third, temporal SCH changes after bed bath appeared to differ between the lower limbs and forearms; however, the mechanism underlying this difference remains unclear. Fourth, the lower limb is not the recommended site for measuring the skin barrier function [43]. Compared to the forearm, the lower limb has body hair and an uneven surface. In this study, we minimized the influence of these factors on the measurements by selecting areas of the lower limbs with little body hair and properly positioning the participants. However, because the lower limbs have less subcutaneous fat and muscle mass, it was more difficult to keep the probe in stable contact with the skin compared with the forearm. Therefore, the difficulty in measuring skin barrier function on the lower limbs may have contributed to the differences observed in the temporal changes in the skin barrier function between the forearms and lower limbs after bed bathing. We fully acknowledged this limitation and implemented appropriate measures to ensure accurate assessment of the skin barrier function, along with confirming the reliability of the measurements using the ICC. Finally, as only one type of disposable towel was used in this study, future research should assess whether similar outcomes can be achieved using other types of disposable towels.

## Conclusion

This study examined the effects of a single bed bath with weak wiping pressure using cotton and disposable towels on the skin barrier function of the lower limbs and forearms in older hospitalized patients with heart disease. Changes in TEWL and SCH over time revealed that a single bed bath with weak wiping pressure did not cause sustained impairment of the skin barrier function by the following day. The ODS, an indicator of skin dryness, did not change between the day before and day after a bed bath. The same trend was observed over time for the skin barrier function of the lower limbs after bed bath in patients with CKD and dry skin who had heart disease, who were at higher risk of skin barrier dysfunction. However, because a temporary decrease in SCH was observed on the forearm, and data are lacking for patients without heart disease, repeated interventions, and changes in the skin barrier function beyond the following day, further studies are needed to clarify the underlying mechanisms.

## Supplementary Information

Additional file 1. TREND statement checklist.

## Data Availability

The datasets used and/or analyzed in the current study are available from the corresponding author upon reasonable request.

## Abbreviations

BMI: Body mass index
CKD: Chronic kidney disease
CONUT: Controlling nutritional status
ICC: Intraclass correlation coefficient
MMRM: Mixed-effects models for repeated measures
ODS: Overall dry skin score
SCH: Stratum corneum hydration
TEWL: Transepidermal water loss
